# A case report of recurrent primary posterior mediastinal perivascular epithelioid cell tumour compressing the right inferior pulmonary vein, atria, and inferior vena cava

**DOI:** 10.1093/ehjcr/ytae142

**Published:** 2024-03-20

**Authors:** Preetham Kumar, Nolan S Maloney, Anees Razzouk, Ramdas G Pai, Padmini Varadarajan

**Affiliations:** Division of Cardiology, Department of Medicine, University of California, Riverside, 900 University Ave, Riverside, CA 92521, USA; Department of Pathology, Loma Linda University Medical Center, Loma Linda, CA, USA; Department of Cardiothoracic Surgery, Loma Linda University Medical Center, Loma Linda, CA, USA; Division of Cardiology, Department of Medicine, University of California, Riverside, 900 University Ave, Riverside, CA 92521, USA; Division of Cardiology, Department of Medicine, University of California, Riverside, 900 University Ave, Riverside, CA 92521, USA

**Keywords:** Cancer, Case report, Cardiac magnetic resonance, Chest pain, Echocardiography, Imaging, Shortness of breath

## Abstract

**Background:**

Perivascular epithelioid cell tumours (PEComas) are rare soft tissue neoplasms that commonly occur in the uterus, skin, and liver and less commonly in the retroperitoneum, colon, and mediastinum.

**Case summary:**

A 36-year-old male patient with a history of mediastinal PEComa status post resection, essential hypertension, and atrial fibrillation status post appendage ligation currently not on anticoagulation presented with a 1-week history of fevers, chills, productive cough, chest pain, dyspnoea on exertion, loss of appetite, and general weakness. Vital signs, physical exam, laboratory data, electrocardiogram, and chest radiograph were grossly unremarkable. A multimodality imaging approach utilizing transthoracic echocardiogram, transoesophageal echocardiogram (TEE), cardiac magnetic resonance imaging (cMRI), and computed tomography angiography of the chest, abdomen, and pelvis revealed a local 40 mm × 53 mm globular bilobed vascularized scar-free posterior mediastinal mass arising from the roof of the left and right atria and extending superiorly to the main pulmonary artery and inferiorly to the inferior vena cava. Based on the mass’ size and proximity to vital structures and tumour recurrence, the case was presented during tumour board rounds, and the outcome was to surgically resect the mass and then have the patient follow up with medical oncology and radiation oncology for possible chemotherapy and radiation, respectively.

**Discussion:**

Perivascular epithelioid cell tumours are rare, and mediastinal PEComas are even rarer, warranting a multimodality imaging approach involving TEE and cMRI and a multidisciplinary approach involving anaesthesiologists, cardiologists, cardiothoracic surgeons, medical oncologists, pathologists, radiologists, and radiation oncologists.

**Learning points**

- To understand the clinical presentation, diagnosis, and management of recurrent posterior mediastinal PEComas.
- Demonstrate the value of multimodality imaging and multidisciplinary collaboration among specialists from a variety of disciplines in the setting of challenging diagnoses.
- To be aware that primary PEComas can infrequently lack expression for melanocytic markers.

## Introduction

Perivascular epithelioid cell tumours, or PEComas, are rare mesenchymal neoplasms composed of histologically and immunohistochemically distinctive perivascular epithelioid cells that are usually immunoreactive for both smooth muscle and melanocytic markers.^1^ The most common neoplasms in the PEComa family are renal angiomyolipoma and pulmonary lymphangioleiomyomatosis, but PEComas can arise in soft tissue as well. To date, there are an ample number of case reports describing anterior mediastinal PEComas, either as primary tumours or secondary to metastasis, and a scarce amount related to metastatic posterior mediastinal PEComas, but none related to primary posterior mediastinal PEComas, highlighting the extreme rarity of the presenting condition. Here, we report an adult patient with a symptomatic posterior mediastinal PEComa, which was successfully surgically repaired.

## Summary figure

**Figure:**
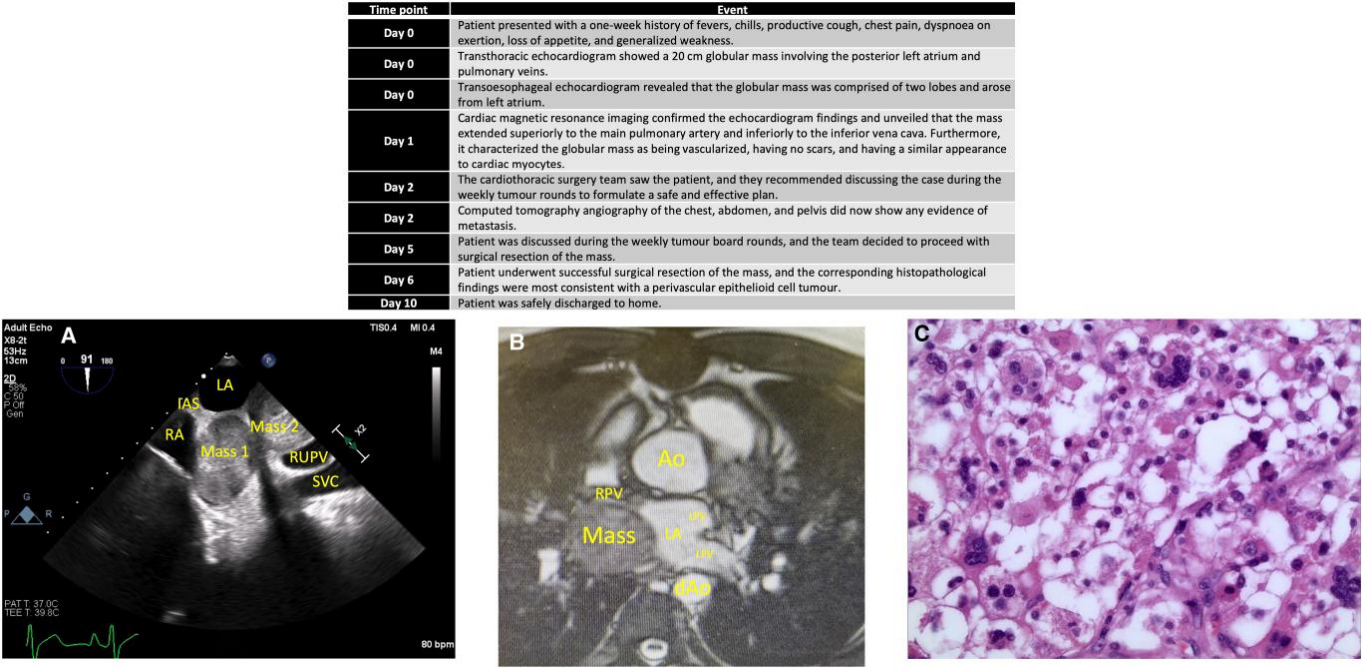


## Case presentation

A 36-year-old male presented with a 1-week history of fevers, chills, productive cough, chest pain, dyspnoea on exertion, loss of appetite, and generalized weakness. The chest pain was described as being located in the middle of the back, pressure like in quality, constant in duration, 5/10 in severity, non-radiating, and non-pleuritic.

The patient’s history is notable for mediastinal PEComa status post resection, essential hypertension, and paroxysmal atrial fibrillation status post appendage ligation currently not on anticoagulation. Of note, the initial occurrence of PEComa was originally misdiagnosed as a rhabdomyoma, and it was correctly identified as a PEComa upon re-evaluation of the corresponding histopathological slides during the current presentation. Since the evaluation and management of the first presentation occurred at an outside facility, details regarding the final diagnosis are limited. Additionally, indication for appendage ligation, performed at the same time the PEComa was resected, is also unclear, but since the patient is young and has a low CHA_2_DS_2_-VASc score of 1, the most likely reason is being off lifelong anticoagulation.

Given the patient had a history of a mediastinal PEComa, tumour recurrence was highest in the differential. Other aetiologies included pneumonia.

Vital signs were unremarkable. Physical exam was notable for an irregularly irregular rhythm and sternotomy scar. Laboratory data, including inflammatory markers, were unremarkable. Electrocardiogram was significant for normal sinus rhythm, first-degree atrioventricular block, and left atrial enlargement. Chest radiograph was unremarkable.

Based on concerns for tumour recurrence, a transthoracic echocardiogram was obtained, and it showed a globular mass involving the posterior left atrium and pulmonary veins and measuring 20 cm circumferentially.

**Figure 1:**
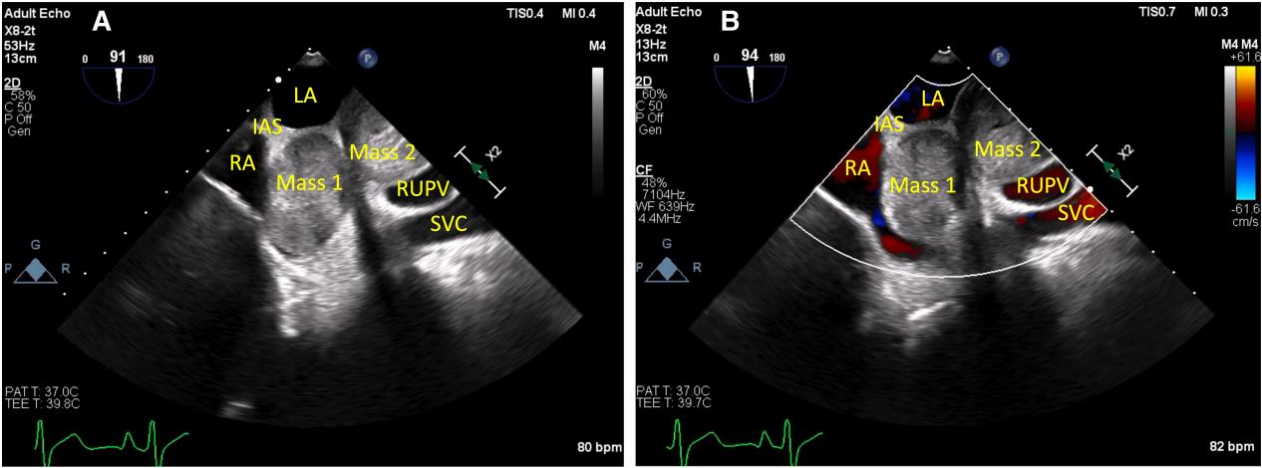
(*A*) Transoesophageal echocardiogram with (*B*) colour flow Doppler showing a large globular bilobed mass arising from the posterior aspect of the left atrium, (*A*) pushing onto the interatrial septum, (*B*) the posterior left atrium, and right pulmonary vein. IAS, interatrial septum; LA, left atrium; RA, right atrium; RUPV, right upper pulmonary vein; SVC, superior vena cava.

Since the mass appeared to involve posterior cardiac structures, and transoesophageal echocardiography, compared with transthoracic echocardiography, offers better visualization of posterior cardiac structures due to close proximity of the oesophagus to the posteromedial heart with lack of intervening lung and bone, a transoesophageal echocardiogram was performed. The echocardiogram showed a large globular bilobed mass arising from the posterior aspect of the left atrium near the roof, pushing onto the interatrial septum into the right atrium, and either extending into or arising from the right upper pulmonary vein, with one lobe measuring 36 mm × 36 mm (*Figure 1*).

**Figure 2:**
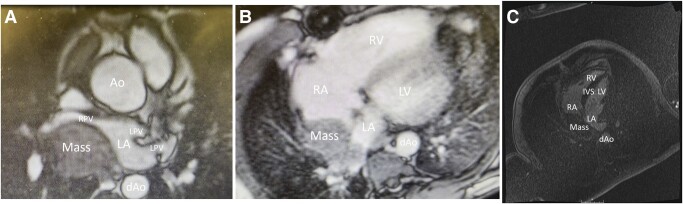
(*A*) Steady-state free precession cardiac magnetic resonance imaging showing the mass compressing the posterior left atrium and right pulmonary vein. (*B*) First-pass perfusion cardiac magnetic resonance imaging showing enhancement of the mass, suggesting hypervascularity. (*C*) Delayed enhancement cardiac magnetic resonance imaging signifying the presence of a rich stroma and neovascularized mass. Ao, aorta; dAo, descending aorta; IVS, interventricular septum; LA, left atrium; LPV, left pulmonary vein; LV, left ventricle; RA, right atrium; RPV, right pulmonary vein; RV, right ventricle.

Given ambiguity about structures affected by the mass as well as features of the mass, a cardiac magnetic resonance imaging (cMRI), which offers high spatial and temporal resolution, especially compared with ultrasound, and the ability to characterize tissue, was ordered. The cMRI showed a 40 mm × 53 mm bilobed posterior mediastinal mass abutting the roof of the left atrium and right atrium, compressing the right inferior pulmonary vein, and extending superiorly to the main pulmonary artery and inferiorly to the inferior vena cava (*Figure 2*). T_1_-weighted images demonstrated that the mass had a similar appearance to cardiac myocytes. T_2_-weighted images demonstrated that the mass was hyperintense to skeletal muscle. First-pass perfusion signal was elevated, highlighting that the mass was hypervascularized. Delayed enhancement was present, reflecting the rich stroma and neovascularization of the mass. Inversion recovery sequencing revealed no scars. These findings, together, suggested recurrent PEComa, thymoma, teratoma, or leiomyoma. Furthermore, the imaging features, specifically a well-defined mass without invasion of adjacent cardiac structures and pericardial effusion/thickening, favoured a localized lesion over a metastatic one.

To stage the cardiac mass, a computed tomography angiography of the chest, abdomen, and pelvis was performed, and it re-demonstrated the mass without any evidence of metastasis.

Cardiothoracic surgery (CTS) was consulted, who, upon thorough review of all available data and after discussing the case during weekly tumour board rounds, recommended surgical resection of the mass given its large size, invasive features (i.e. compressing the right inferior pulmonary vein, right and left atria, and inferior vena cava), and proximity to the oesophagus. Of note, analysis of the operative report related to resection of the initial mediastinal PEComa revealed that the initial mass and recurrent mass originated from the same location.

Since the patient had the prior mass resected via a median sternotomy, the repeat resection was performed via a right lateral posterior thoracotomy while on cardiopulmonary bypass using peripheral cannulation. Furthermore, hypothermia was induced so the heart could be safely fibrillated, allowing for reconstruction of the pulmonary vein and left atrium without arresting the heart. Intraoperative findings were consistent with imaging findings.

Patient had an uncomplicated recovery course, and he was safely discharged home on postoperative Day 6.

**Figure 3:**
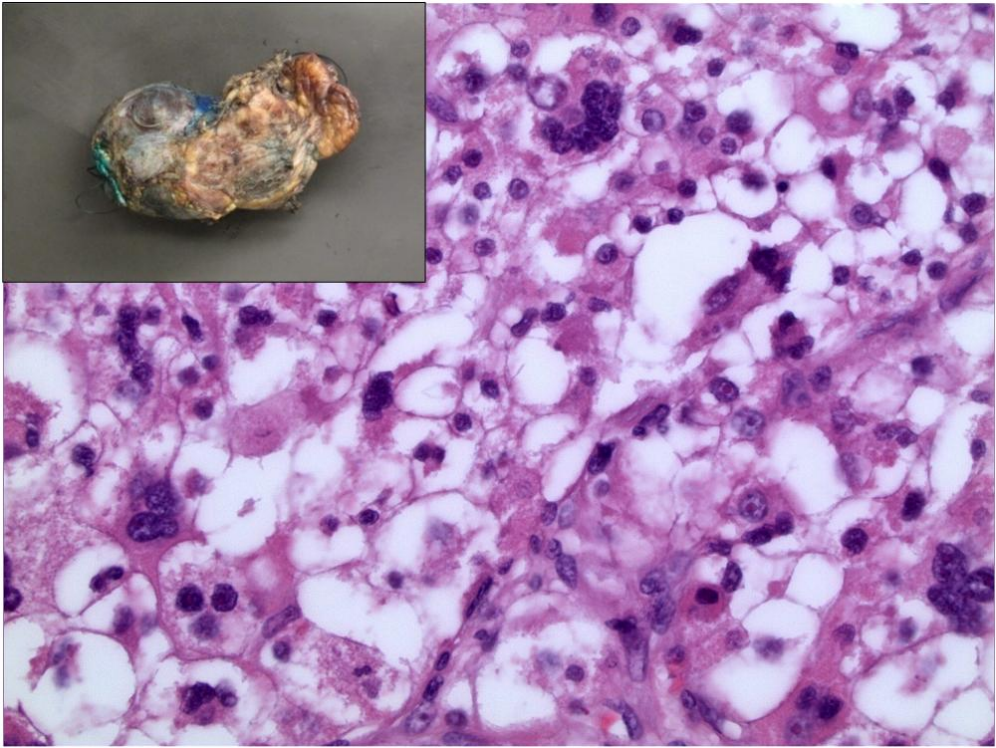
Haematoxylin and eosin staining at ×400 demonstrating features compatible with a malignant perivascular epithelioid cell tumour, notably epithelioid cells with clear to densely eosinophilic cytoplasm and scattered marked nuclear pleomorphism. *Inset*: Gross external view demonstrating the irregularly shaped tan mass.

Pathologic examination of this tumour revealed a portion of intact tumour that was partially encapsulated with areas of capsular disruption and a separate aggregate of tumour fragments (*Figure 3*). The cut surface of the tumour was tan, firm, and slightly friable in areas with focal areas of haemorrhage. Microscopic examination showed tumour cells to have an epithelioid morphology with abundant clear to eosinophilic cytoplasm and scattered prominent nuclear atypia (*Figure 3*). Immunohistochemical stains highlighting myogenic differentiation, such as smooth muscle actin and desmin, were positive, and the proliferation index by ki-67 was 20–30%; no definite melanocytic differentiation was identified. No definite invasion of the myocardium was identified. In summary, despite the lack of definitive melanocytic differentiation, these findings were most consistent with a malignant PEComa. Due to the piecemeal nature of the resection, definite evaluation of margins was not possible.

Following discussion of the case and final pathology report during tumour board rounds, the healthcare team believed it was in the best interest of the patient to follow-up with medical oncology and radiology oncology for further recommendations, including initiation of everolimus and radiation, respectively.

Patient was seen in CTS clinic ∼1 month after he was discharged from the hospital, and he reported doing well, including complete alleviation of all the original presenting symptoms. Additionally, the pathology report and recommendations were shared, and patient acknowledged both.

## Discussion

This is the first case report, to the best of our knowledge, of a recurrent primary posterior mediastinal PEComa. It demonstrates the potential challenges of making a diagnosis when the presentation is atypical, since it is uncommon for PEComas to lack definitive evidence of melanocytic differentiation, leading to misdiagnosis, as in this instance. Lastly, this report highlights the utility of multimodality imaging and a multidisciplinary approach for optimal diagnosis, management, and outcome, especially in the setting of challenging diagnoses.

Given the extreme rarity of PEComas and lack of data regarding their natural history and prognostic features, Folpe *et al*.^2^ presented their experience with 26 PEComas of soft tissue and the gynaecologic tract origin. Clinically, 85% (22/26) involved females. Microscopically, 38% (10/26) demonstrated a mixture of epithelioid and spindled cells, 58% (15/26) displayed intermediate nuclear grade, 69% exhibited moderate cellularity (18/26), 69% (18/26) contained giant cells, and 31% (8/26) had evidence of haemorrhage and/or necrosis. Histopathologically, 100% stained positive for at least one smooth muscle marker and at least one melanocytic marker. Similarly, Bao *et al*.^3^ analysed the histopathologic characteristics and immunotypes of 26 PEComas. Clinically, 85% (22/26) involved females, 92% (24/26) involved the kidneys, 100% (26/26) were primary tumours, and 50% (13/26) were invasive. Microscopically, 35% (9/26) demonstrated an epithelioid cell type, 77% (20/26) showed a scattered polymorphism, 14/26 (54%) contained giant cells, and 10/26 (38%) had evidence of haemorrhage and/or necrosis. Histopathologically, 100% stained strongly positive for a smooth muscle marker, either smooth muscle actin, calponin, or vimentin, and 100% tested moderately to weakly positive for a melanocytic marker, either HMB-45, melan A, or desmin.

In contrast, our patient’s PEComa occurred in a male, involved the posterior mediastinum, was a primary tumour, demonstrated an epitheloid cell type, stained positive for the smooth muscle marker SMA, and did not stain positive for any melanocytic markers. Given some of the atypical features, additional input was sought after from a second pathologist, who ultimately agreed with the diagnosis of a PEComa, commenting that melanocytic markers, though commonly expressed by such tumours, can be absent, and close attention must be paid to the morphological features for an accurate diagnosis.

This case report also highlights the advantages of cMRI, an advanced imaging modality, in the setting of uncertain diagnoses. In this instance, the use of different cMRI sequences helped clarify the anatomy and appearance and presence of scarring and vascularity. Current guidelines recommend cMRI to augment other findings in the setting of intra- and extra-cardiac tumours given its benefits of multiplanar image acquisition, high spatial resolution, large field of view, and tissue characterization.^4^

Lastly, this case report emphasizes the importance of a multidisciplinary approach in the setting of challenging diagnoses. Once a diagnosis of a posterior mediastinal mass was made, CTS was promptly consulted, who discussed the case with other CTS and in the weekly tumour board rounds prior to offering surgery to ensure it was the best option. Furthermore, the pathologist, upon finishing the workup, sought the opinion of a second pathologist to ensure the final diagnosis was accurate prior to making any comments. These actions undermine the difficulty and novelty of the presenting case.

## Conclusion

Although primary posterior mediastinal PEComas are very rare, they should be in the differential pending final pathology report. During instances of uncertain presentations, multimodality imaging and multidisciplinary approach are critical for accurate diagnosis and optimal outcomes.

## Data Availability

Data are available upon request.

## Lead author biography

Preetham Kumar is currently completing his General Cardiology Fellowship at the University of California, Riverside. He earned his Bachelor of Science in Biomedical Engineering: Premedical at the University of California, Irvine, and his Doctor of Medicine at Virginia Commonwealth University, and he completed his Internal Medicine Residency at Huntington Hospital.

**Consent:** The authors confirm that written consent for submission and publication of this case report, including images and associated text, has been obtained from the patient in line with COPE guidance.

**Funding:** None declared.
